# Morin Protects Human Respiratory Cells from PM_2.5_ Induced Genotoxicity by Mitigating ROS and Reverting Altered miRNA Expression

**DOI:** 10.3390/ijerph16132389

**Published:** 2019-07-05

**Authors:** Indhumathi Veerappan, Senthil Kumar Sankareswaran, Rajaguru Palanisamy

**Affiliations:** Department of Biotechnology, Anna University, BIT Campus, Tiruchirappalli 620 024, India

**Keywords:** Particulate matter, cytotoxicity, genotoxicity, comet assay, oxidative stress, miRNA, morin

## Abstract

Chronic fine particulate matter (PM_2.5_) exposure causes oxidative stress and leads to many diseases in human like respiratory and cardiovascular disorders, and lung cancer. It is known that toxic responses elicited by PM_2.5_ particles depend on its physical and chemical characteristics that are greatly influenced by the source. Dietary polyphenolic compounds that possess antioxidant and free radical scavenging properties could be used for therapeutic or preventive approaches against air pollution related health hazards. This study evaluates characteristics and toxicity of PM_2.5_ collected from rural, urban, industrial, and traffic regions in and around Coimbatore City, Tamilnadu, India. Traffic PM_2.5_ particles contained higher amounts of metals and polycyclic aromatic hydrocarbons (PAHs). It also possessed higher levels of oxidative potential, induced more intracellular reactive oxygen species (ROS), and caused more levels of cell death and DNA damage in human respiratory cells. Its exposure up regulated DNA damage response related miR222, miR210, miR101, miR34a, and miR93 and MycN and suppressed Rad52. Pre-treatment with morin significantly decreased the PM_2.5_ induced toxicity and conferred protection against PM_2.5_ induced altered miRNA expression. Results of this study showed that cytoprotective effect of morin is due to its antioxidative and free radical scavenging activity.

## 1. Introduction

Environmental pollution has become the major concern over human health in recent years. According to World Health Organization, air pollution was found to be the reason for the death of nearly 4.2 million people worldwide in 2016. Urban people are more exposed to the ambient particulate matters (PM) correlating to the exponentially increasing usage of vehicles and industrialization [1]. It is estimated that nearly 91% of urban people are exposed to particulate matters exceeding the WHO guided standards in 2016 [2]. Majorly, PM are emitted by fuel combustion in vehicles, power plants, industries, households and also from biomass burning [3]. Fine particulate matter (PM_2.5_) are mainly responsible for serious health hazards in human including lung cancer, COPD (Chronic Obstructive Pulmonary Disorder) and cardiovascular diseases as it penetrates into deeper parts of the respiratory system including alveoli [4,5]. Factors that decide the toxicity of the PM_2.5_ include their size, surface area, number distribution, chemical composition, and proinflammatory and oxidative properties [6,7]. Presence of transition metals, polycyclic aromatic hydrocarbons (PAHs), and their oxygenated derivatives and endotoxins in PM_2.5_ induce cytotoxicity, inflammation, oxidative stress, and DNA damage [6,8,9,10,11,12].

PM_2.5_ exposure activates the leukocytes and causes inflammation. Inflammation leads to the release of proinflammatory cytokines, reactive oxygen species, and reactive nitrogen species, and cause oxidative stress [13,14,15]. PM_2.5_ themselves can cause oxidative stress on prolonged exposure since they have higher levels of oxidative potential [16,17]. Oxidative stress is a potent inducer of DNA damage. Hence PM_2.5_ exposure can potently induce DNA damage either directly or through the PM_2.5_ induced oxidative stress [18,19,20]. So PM_2.5_ exposure can cause toxicity by either one of the three mechanisms or by the combined effect of two or all mechanisms.

Cellular stressors including environmental toxicants and radiation had been linked to carcinogenesis and altered miRNA expression. Exposure to metal rich PM increased the expression of three oxidative stress and inflammation associated miRNAs; miR222, miR21, and miR146a in leukocytes [21]. A study on the effect of diesel exhaust particles revealed altered expression of 197 miRNAs in human airway epithelial cells [22]. Pre-natal exposure to PM pollution induced significant alterations in the expression of several DNA damage response related miRNAs such as miR21, miR222, miR146a, and miR20a with different magnitudes depending on the trimester and amount of exposure [23]. In the cells under oxidative stress, significant alterations were observed in miR210 miR21, miR34 family, miR200 family, miR146a, miR210, and miR let-7 family (Dando I et al., 2015 [24,25] Increased miR210 expression was found to up regulate the expression and activity of antioxidant enzymes CAD, SOD, GSH-Px [24]. MiR34a, miR93, and miR200a have also been reported to activate antioxidant mechanism through regulation of Nrf2 [26,27]. Exposure to arsenic, sulphates of iron, and aluminum significantly altered the expression of oxidative stress, inflammation and DNA damage related miRNAs including miR210, miR34a, miR21, miR221 and miR222, miR9, miR125b, and miR128 [28,29].

Recently, research on the naturally derived polyphenols has escalated to newer heights as protective and preventive therapeutics unveiled their intervention in various physiological processes by activating several signaling pathways. Modulation of PM_2.5_ induced pathology, using dietary polyphenolic compounds could reveal a novel therapeutic or preventive approach against the air pollution related health hazards [30,31]. Epigallocatechin-3-gallate has been reported to restrain lung cancer through up regulation of miR210 via reduced ubiquitination, thus stabilization of the pro-angiogenic factor HIF1α [32]. A study on beneficial effect of seven chemopreventive agents including two dietary agents (Phenethyl isothiocyanate and Indole-3-carbinol) revealed the ability of the agents to revive the cigarette smoke induced alteration in expression of miRNAs involved in cancer related cellular mechanisms like p53 activity, NFκB activation, cell proliferation, and apoptosis [33].

Morin is a potent antioxidant with high free radical scavenging activity and found in higher concentrations in white mulberry and other plants of Moracea family. Morin has also been showed to significantly reduce the amount of ROS production during oxidative stress induced at various instances [34,35]. Morin has been observed to modulate the Nrf2 signaling pathway [36,37] and reported to suppress the expression of several miRNAs such as miR155, miR330, and miR135b [38,39]. These studies suggest the ability of morin to influence the expression of miRNAs and its use to conferring protection over PM_2.5_ induced toxicity. This study mainly focuses to reveal the protective nature of morin over the PM_2.5_-induced genotoxicity by decreasing oxidative stress and altering miRNA expression. For this study, PM_2.5_ samples were collected from Coimbatore, Tamilnadu state, India since it is the major focal point of industries, heavy traffic and high population. It is one of the fastest-growing tier II cities in India with nearly 25,000 textile and engineering industries [40]. Coimbatore is long recognized for the abundance of textile industries, motor and pump manufacturing industries and associated foundries. The city consists of six main heavy traffic roads including five national highways connecting important cities of South India and also many connecting feeder roads [41]. The industrial and traffic hub also makes the city most vulnerable for air pollution.

## 2. Materials and Methods

### 2.1. Collection and Extraction of PM_2.5_

The PM of size less than 2.5 µm (PM_2.5_) were collected from multiple locations of different background (Urban-11.0266° N, 77.0212° E, Industrial-11.0494° N, 77.0094° E, Traffic-10.9951° N, 76.9594° E, Rural/Agricultural-10.9899° N, 76.8409° E) from May to September, 2016. No rainfall occurred on all sampling days and the wind flow was mainly from Southwest direction with velocity ranging between 5.52 and 16.71 km/h. At least 15 samples were collected at each location for 8 h during day time using air sampling devises with cyclone separator and Teflon Filter for PM_2.5_ (Envirotech APM550, India) in Coimbatore, Tamilnadu State, India. Metals and Poly aromatic hydrocarbons (PAHs) were extracted from separate sets of at least 4 filters collected at each sampling location. Metals were extracted using acid digestion and PAHs were extracted ultrasonically using dichloromethane as described by Senthil kumar et al. (2014) [6]. PM_2.5_ were extracted from another set of 4 filters by soaking them in 10 mL of double distilled water and sonicated for 15 min. The supernatants were collected and dried over hotplate at 50 °C. PM_2.5_ collected from four samples at each location were pooled and used further.

### 2.2. Characterization of PM_2.5_

The average hydrodynamic size and zeta potential of PM_2.5_ samples in water were determined by dynamic light scattering (Nano-Zeta Sizer-HT, Malvern Instruments, Malvern, PA, USA).Metals and PAHs in PM were extracted and measured as described by Senthil kumar et al. (2014) [6]. The samples were analysed for the presence of 12 metals (As, Cd, Cu, Cr, Fe, Mn, Ni, Zn, Se, Al, V, and Pb) using AAS (Perkin Elmer Analyst 300, USA) and for 16 PAHs including Napthalene (NaP), Acenapthylene (Acy), Acenapthene (Ace), Fluorene (Fl), Phenanthrene (Phe), Anthracene (Ant), Fluoranthene (Flu), Pyrene (Pyr), Benzo(a)anthracene (BaA), Chrysene (Chr), Benzo(b)fluoranthene (BbF), Benzo(k)fluoranthene (BkF), Benzo(a)pyrene (BaP), Dibenzo(a,h)anthracene (DBA), Benzo(ghi)pyrene (BghiP), and Indeno(123-cd)pyrene (IND) using HPLC-fluorescence (Shimatzu 10A, Kyoto, Japan) and a standard reference material (Aqua Standard, USA) as described by Senthil kumar et al. (2014) [6].

### 2.3. Estimation of Endotoxin

The presence of endotoxin in the samples was determined by Limulus Amebocyte assay (Kinetic Turbidimetric LAL Assay, Lonza, Basel, Switzerland). All samples were analysed in triplicates at 100 µg/mL concentration as described by Shalini et al. (2018) [42].

### 2.4. Estimation of Oxidative Potential of PM_2.5_

The oxidative potential of the PM_2.5_ samples was estimated using the Dithiothreitol (DTT) assay [43]. The PM_2.5_ samples (25 and 50 µg/mL) suspended in 0.5 M phosphate buffered saline (PBS, pH 7.4) were incubated with 0.1 M DTT solution for 0–45 min at 37 °C. Prior to every assay the stock suspension (1000 µg/mL) of PM_2.5_ was sonicated for 30 s three times. After incubation an aliquot of the reaction mixture was mixed with 10% trichloroacetic acid and then mixed with Tris buffer (pH 8.9), 20 mM Ethylenediamine tetra aceticacid (EDTA) and 10 mM 5,5′-dithio-bis-(2-nitrobenzoic acid) (DTNB) solution. The formation of the resultant 5-mercapto-2-nitrobenzoic acid was measured at 412 nm. The oxidative potential of the PM_2.5_ sample is expressed as the rate of DTT consumption in nmoles per min of incubation per µg of sample added minus DTT consumption by the control sample. Reaction mixture without PM_2.5_ was used as the control. All treatments were performed in triplicates.

### 2.5. Cell Line and Culture Conditions

Human small cell lung carcinoma (A549) cell line was acquired from NCCS (National Centre for Cell Science, Pune, India) and cultured in DMEM (GIBCO, Invitrogen, USA), supplemented with 10% heat inactivated FBS, 2 mM L-glutamine (GIBCO, Invitrogen, USA) and 100 U/mL penicillin-streptomycin (GIBCO, Invitrogen).

### 2.6. Estimation of Lipid Peroxidation

The effect of PM_2.5_ to induce lipid peroxidation was evaluated by measuring thiobarbituric acid-reactive species (TBARS) (Nichens Niehaus and Samuelsson 1968) [44] and lipid hydroperoxides (LOOH) (Jiang et al. 1992) [45]. Overnight triplicate cultures of A549 cells (5 × 10^4^ cells/well in 24-well plates, at 37 °C), after 1 h exposure to PM_2.5_ (25, 50, and 100 µg/mL) suspended in serum free DMEM were try psinized, centrifuged and the supernatant was removed. The cells were then re-suspended in de-ionized water and sonicated for 20 s (VCX750, Sonics & Materials Inc., Newtown, CT, USA). The cell lysate was used for lipid peroxidation assays as described by Shalini et al., 2014 [42].

### 2.7. Estimation of Cytotoxicity of PM_2.5_

Cytotoxicity of PM_2.5_ was estimated through the lactate dehydrogenase release assay (LDH) and MTT assay. Briefly, overnight cultures of A549 cells (1 × 10^4^ cells/well in 96 well plates, at 37 °C) were treated with 12.5, 25, 50, 100, 200, and 400 µg/mL of PM_2.5_ suspended in serum free DMEM. At each concentration at least 6 replicates were maintained for all the samples. After 24 h, LDH was estimated in the medium as described by Shalini et al. [46] and the cells were used for MTT assay as described by Senthil Kumar et al. [6].

### 2.8. Estimation of Genotoxicity of PM_2.5_

Genotoxicity of PM_2.5_ was elucidated through the Alkaline Comet assay. Overnight culture of A549 cells (1 × 10^5^ cells/well) seeded in 24-well plates were treated with 12.5, 25, and 50 µg/ml of PM_2.5_ suspended in serum free DMEM in triplicates for 24 h at 37 °C. For the comet assay, H_2_O_2_ (100 µM, 5 min, 37 °C) exposed cells were used as positive control. Then the cells were trypsinized and subjected for the comet assay under alkaline conditions following the procedure of Senthil Kumar et al. [6]. Ethidium bromide (10 µg/mL) stained slides were examined at 20× using a fluorescence microscope. A total of 100 randomly selected cells from two replicate slides (50 cells per slide) were examined per sample. Triplicate cultures and controls were maintained for all the samples. The % tail DNA was measured using Comet Score Version 1.5 software and used in all comparisons.

The protective role of morin over PM_2.5_ induced genotoxicity was measured by treating the A549 cells (5 × 10^4^ cells/well in 24-well plates) with 10µM morin in DMSO for 2 h at 37 °C followed by exposure to PM_2.5_ (25 and 50 µg/mL) suspended in serum free DMEM for 4 h at 37 °C. Triplicate cultures and controls were maintained for all the samples.

### 2.9. Measurement of Intracellular Reactive Oxygen Species

Measurement of intracellular reactive oxygen species (ROS) levels was determined by using DCFH-DA dye. The A549 cells (5 × 10^4^ cells/well) taken in 24-well plate were treated with 25 and 50 µg/mL of PM_2.5_ suspended in serum free DMEM for 4 h at 37 °C in triplicates and assayed for ROS levels as described by Shalini et al. (2018) [42]. Separate sets of cells were pre-treated with 10 µM morin in DMSO for 2 h prior to PM_2.5_ treatment to determine the effect of morin over ROS generation. H_2_O_2_ (200 mM, 5 min) was used as the positive control.

### 2.10. Effect of PM_2.5_ and Morin on miRNA and Gene Expression

Overnight triplicate cultures of A549 cells (1 × 10^6^ cells/well in 6-well plate) were treated with traffic PM_2.5_ samples (50 µg/mL) suspended in serum free DMEM for 24-h at 37 °C. For morin pre-treatment, the cells were treated for 2 h with 10 µM morin in DMSO prior to PM_2.5_ exposure. After treatment, total miRNA was isolated using Qiagen miRNeasy Mini kit as per manufacturer’s protocol (Qiagen, Germantown, MD, USA)and subjected to PolyA tailing (Epicentre kit, Illumina, San Diego, CA, USA), followed by poly T conversion to synthesis of cDNA using 2 µg of the total miRNA isolated (Applied Biosystems, Waltham, MA, USA) for further quantification using qRT-PCR with primers enlisted in Table 1. The endogenous control RNU48 was used for normalization. In a separate experiment, total RNA was isolated using the RNeasy kit according to manufacturer’s protocol (Qiagen, Germantown, MD, USA), converted to cDNA (Applied Biosystems, Waltham, MA, USA) and quantified using qRT-PCR with gene specific primers (Table 2) using β-actin as the internal control.

### 2.11. Statistical Analysis

Experimental results were expressed as mean ± SD of three parallel experiments. Data of various parameters were analyzed by Duncan’s multiple range test. The ROS, miRNA and gene expression data were compared using Student’s T test. Values were considered statistically significant when *p* < 0.05.

## 3. Results and Discussion

### 3.1. Characterization of PM_2.5_

PM_2.5_ is the collection of all solid and liquid particles suspended in air; many of which are hazardous; harmful to human health; can lead to cardiovascular diseases, lung disorders, blood marker changes, lung cancer, and also death in humans if exposed to for a prolonged period [46,47,48,49]. In this study the PM_2.5_ collected from four different regions (Rural, Urban, Industrial, and Traffic) in Coimbatore city were characterized and analyzed for their toxicity. The concentration of the PM_2.5_ in all the samples collected from various regions under study violated the WHO standards (PM_2.5_: 20 μg/m^3^ of air, 24 h mean) [50]. The rural samples contained 30.34 μg/m^3^ of air while traffic samples contained 51.0 μg/m^3^ of air. The PM_2.5_ in all the samples were in the sub-micron range from 400 to 2600 nm (Table 3.) and it is well known that the fine particles in this size range enter the respiratory tract, perpetuate in the alveoli and render their toxic effects in the pulmonary tissues [51,52]. All samples had Polydispersity index value of nearly 1.0 verifying the uniform particle size. The rural sample which primarily composed of agricultural wastes and windblown soil particles was found to contain comparably larger particles (759.4 nm) and the industrial sample consisted of smallest particulates (444 nm) which correlates with the fact that particles arising out of high temperature vaporizations, chemical reactions and various other industrial processes are very small [53].

### 3.2. Metals and PAHs

Metals present in PM_2.5_ are majorly responsible for the PM induced toxicity as several metal species are well known to cause cytotoxicity, neurotoxicity, and immunotoxicity, and can lead to several diseases like cancer, cardiopulmonary effects, and hypertension [54,55]. Fe was found to be the most occurring in the ambient air PM_2.5_ samples, with high concentration in the industrial and traffic PM_2.5_ samples (Table 4). The metal dust exhaust from the iron and steel industries and foundries located in and around Coimbatore city could be associated to the abundance of Fe in ambient air. Fe once in environment had been observed to persist for longer duration and add upon to its concentration in air. Excessive inhalation of iron can cause siderosis, anemia and even lung cancer [55]. Next to Fe, Pb was found to be in higher concentration in all the samples. Anthropogenic sources such as combustion of fossil fuels, traffic, metal production, iron and steel industries, and cement factories [56] are considered the major sources for the environmental release of Pb. The mean concentration of Pb was found to be maximum (0.71 ng/m^3^ of air) in traffic air. Cr had been found to be the third most occurring metal in the PM_2.5_ samples, which has been classified as a Class A human carcinogen. Chromium VI ions had been known to cross cell membranes and cause genotoxicity and mutagenic. Steel production, coal and oil burning, stainless-steel welding, and chemical manufacturing had been observed to be the major source of Cr release into the environment [57]. In this study, Cr had been determined to be in highest concentration in the industrial area correlated to the presence of steel industries in the area. The Mn concentration in all the sampling regions had been found to be 0.03 ng/m^3^ of air. All other metals were found to be in trace levels, less than 0.01 ng/m^3^ of air. Endotoxins were found to be absent in all the samples.

The rural PM_2.5_ sample contained the least amount of PAHs (5.6 ng/m^3^ of air) and industrial (25.0 ng/m^3^ of air) and traffic (24.8 ng/m^3^ of air) samples contained higher amounts. Out of 16 PAH for which the sample were tested, traffic sample contained 12, industrial sample contained 8, urban sample contained 7 and rural sample contained 5 PAH compounds. Traffic PM_2.5_ sample contained significant concentrations of Chr (8.2 ng/m^3^ of air), BbF (3.9 ng/m^3^ of air), BaA (2.8 ng/m^3^ of air), and BaP (2.5 ng/m^3^ of air). Three other PHAs, NaP (0.2 ng/m^3^ of air), Acy (0.7 ng/m^3^ of air) and BkF (0.1 ng/m^3^ of air) were detected only in this sample. Highest amounts of Flu (11.5 ng/m^3^ of air), Pyr (4.1 ng/m^3^ of air), Phe (3.6 ng/m^3^ of air), and Ant (3.0 ng/m^3^ of air) were found in the industrial sample. While Flu, BaP, and DBA were detected in all 4 types of samples, BaA, Chr, and BbF were found in traffic and urban samples only. BghiP and IND were not detected in any of the sample (Table 5).

### 3.3. Oxidative Potential of PM_2.5_

**Figure 1:**
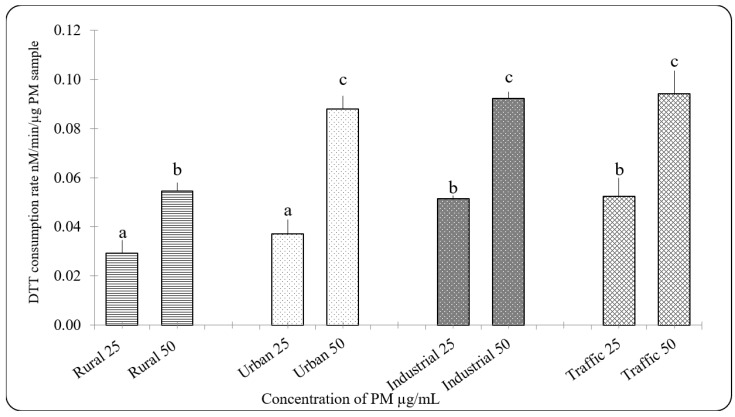
Oxidative potential of PM_2.5_ measured by DTT assay, N = 3 ± SD. Dataset not sharing common superscript vary significantly *p* < 0.05 Duncan’s Multiple Range Test (DMRT).

It had been reported that chronic exposure to PM_2.5_ beyond recommended level instigates protective and detrimental cellular responses via oxidative stress [58]. Oxidative potential of PM_2.5_ has been used as a measure to describe the potential to elicit oxidative stress [59]. Oxidative potential of the PM_2.5_ samples as measured as DTT consumption rate showed that rural sample showed lowest oxidative potential and the traffic sample showed higher oxidative potential than urban and industrial samples (Figure 1).

**Figure 2:**
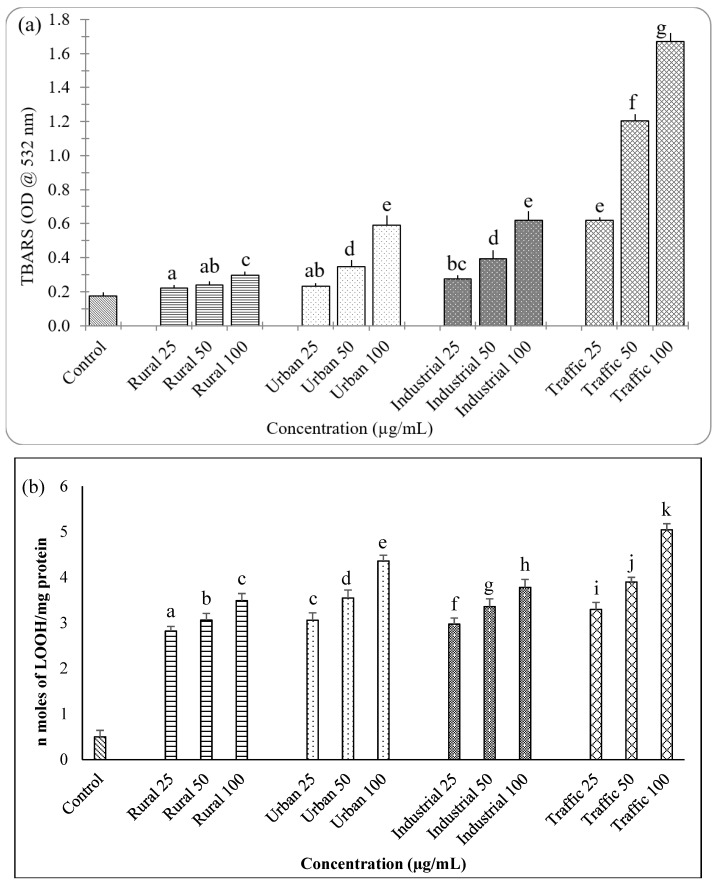
Thiobarbituric acid-reactive species (TBARS) (**a**) and lipid hydroperoxides (LOOH) (**b**) measured in A549 cells treated with PM_2.5_ samples. N = 3 ± SD, Dataset not sharing common superscript vary significantly *p* < 0.05 (DMRT).

The potential of the PM to elicit oxidative stress in cells were studied by measuring lipid peroxidation in A549 cells by TBARS and LOOH assay (Figure 2a,b). In both the assays the PM_2.5_ samples showed increasing levels of lipids peroxidation in a dose dependent manner. The traffic PM_2.5_ sample which had highest level of oxidative potential was found to induce more lipid peroxidation in the cells and the rural sample had least effect. The higher level of lipid peroxidation in traffic PM_2.5_ might be correlated to the higher metal concentration and organic substances than the other three types of samples.

### 3.4. Cytotoxicity and Genetoxicity in A549 Cells

**Figure 3:**
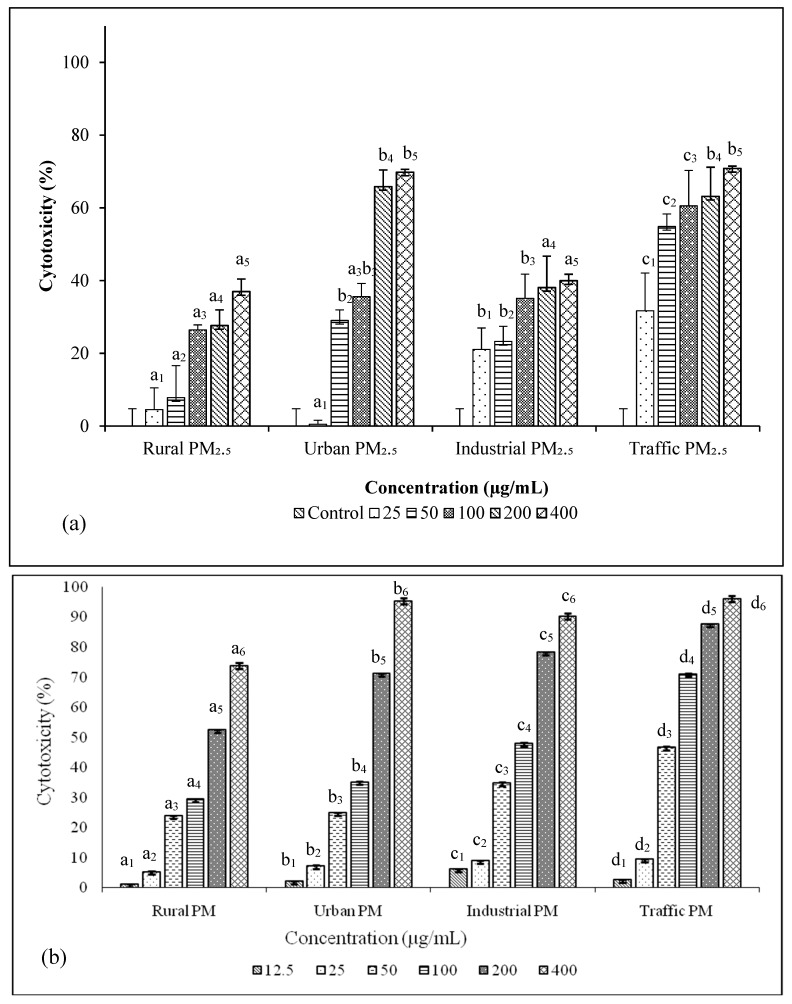
Cytotoxicity of PM_2.5_ samples measured in A549 cells by MTT assay (**a**) and lactate dehydrogenase release assay (LDH) release assay (**b**), N = 6 ± SD. Dataset not sharing common superscript vary significantly *p* < 0.05 (DMRT).

The detrimental effect of PM_2.5_ could be understood on the basis of their ability to induce cytotoxicity and genotoxicity in cells. MTT assay and LDH release assay had been performed to analyze the cytotoxicity of PM and both showed dose dependent increase in cytotoxicity (Figure 3a,d,b) as observed in other studies [60,61]. In MTT assay, IC50 of traffic PM_2.5_ was found to be 50µgand that of urban PM_2.5_ sample was 200 µg/mL. In the case of rural and industrial samples, cell viability was found to be around 60% at 400 µg/mL PM_2.5_ concentration.

While in the LDH release assay, which measures membrane integrity and damage, nearly 50% cell death was found in 200 µg/mL concentration of rural and urban sample, 100 µg/mL of industrial PM_2.5_, and 50 µg/mL of traffic PM_2.5_ sample.

**Figure 4:**
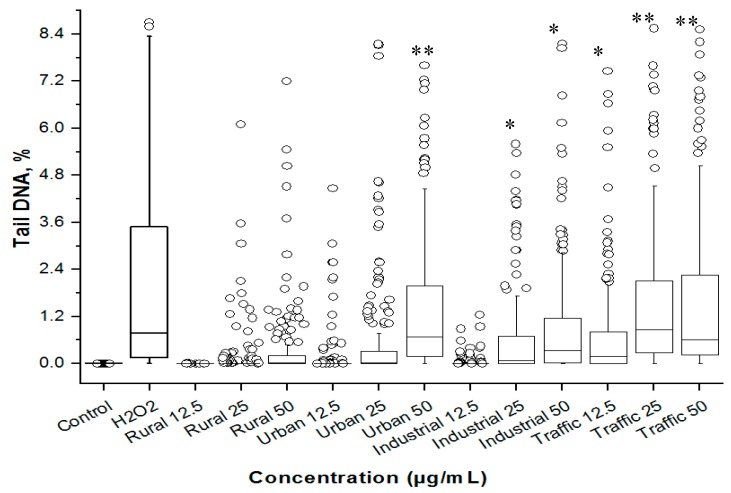
Box-and-whisker plot of the distribution of DNA damage (% tail DNA) in A549 cells treated with PM_2.5_ for 24 h at 37 °C and measured using the alkaline comet assay. The boxes include 50% of the data. The inner line marks the median value and whisker lines extending from the box represent the minimum and maximum values. H_2_O_2_ (100 mM, 5 min at 37 °C) was used as the positive control. Small circles represent outliers. * *p* < 0.05; ** *p* < 0.01.

The PM_2.5_-treated A549 cells were then subjected to the alkaline comet assay to understand their potential to induce genotoxicity (Figure 4). The tail DNA% obtained in the assay clearly revealed the toxicity pattern congruent with the results of the cytotoxicity assays. At all the test concentrations (12.5, 25, and 50 µg/mL), the rural PM_2.5_ sample did not induce any detectable levels of DNA strand breaks (Figure 4). Urban PM_2.5_ samples induced significantly (*p* < 0.01) high levels of DNA damage only at 50 µg/mL dose. Dose dependent increase in DNA damage was observed in industrial and traffic PM_2.5_ samples treated A549 cells. The size of the boxes and position of the median line indicated a dose dependent increase in the number of cells with high levels of DNA damage. The presence of outliers in all treatment groups indicated the occurrence of exceedingly high levels of DNA strand breaks in a small group of cells.

### 3.5. Morin Protects Cells from PM_2.5_ Induced Genotoxicity by Reducing ROS Production

**Figure 5:**
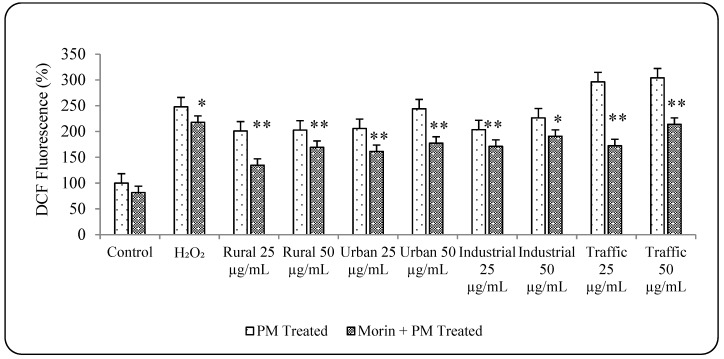
Effect of Morin on PM_2.5_ induced ROS production. N = 3 ± SD, * *p* < 0.05 ** *p* < 0.01.

**Figure 6:**
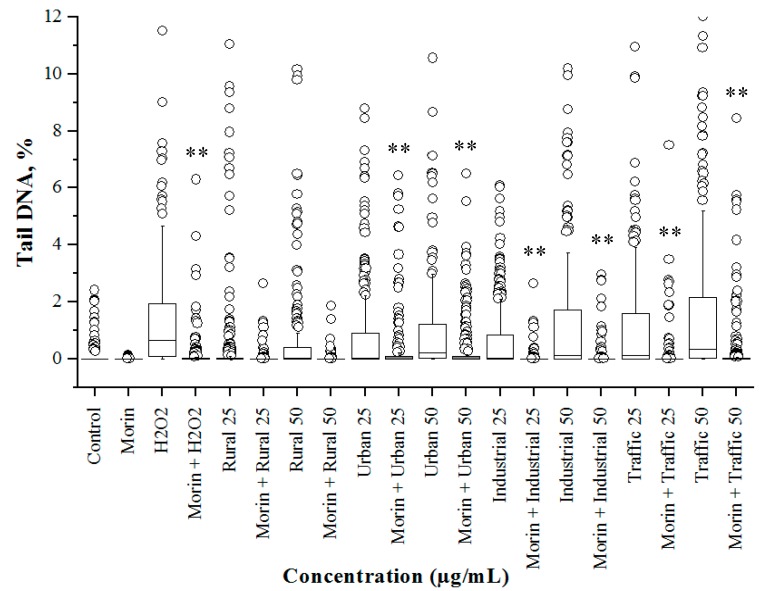
Plot of the distribution of DNA damage (% tail DNA) in morin pretreated (2 h) A549 cells treated with PM_2.5_ for 4 h at 37 °C. The boxes include 50% of the data. The inner line marks the median value and whisker lines extending from the box represent the minimum and maximum values. H_2_O_2_ (100 mM, 5 min at 37 °C) was used as the positive control. Small circles represent outliers. * *p* < 0.05; ** *p* < 0.01.

Morin is a naturally occurring antioxidant scavenging free radicals and thus reducing the ROS production level. It has been shown to compromise the oxidative stress produced in the human cell due to adverse causes, inhibits genotoxicity and prevent the progression of many related diseases [34,35,36,37,62,63,64]. The beneficial effect of morin on reducing the PM_2.5_ induced ROS production and genotoxicity was evaluated in this study in an attempt to establish a prophylactic protection against PM related health hazards in human. Pre-treatment of A549 cells with 10 µM morin effectively reduced the ROS production (Figure 5) and also the tail DNA% in all the PM_2.5_-treated samples. Morin pre-treatment significantly (*p* < 0.01) reduced the DNA damage induced by PM_2.5_ samples (Figure 6). However, existence of large number of outliers in all the treatment groups indicated the incomplete recovery of the cells from the DNA damage caused by PM_2.5_ samples. Thus, it could substantiate the protective effect of morin over PM induced toxicity by reducing the oxidative stress.

### 3.6. PM_2.5_ from Traffic Region Confers More Toxicity

It can be understood that the types and nature of the PM_2.5_ will differ according to the source of the sampling regions [8] and it is also a known fact that physical and chemical characteristics of the particles play an important role in eliciting a toxic response [6,42]. A comparative study on the effect of walking in traffic area and non-traffic area in London among elderly adults reported that the beneficial improvement in lung function by walking observed in non-traffic area had been curtailed in the traffic area showing the intensity of traffic related pollution [65]. Histone modifications such as acetylation and methylation had been found to be occurring most among the truck drivers than the office workers that could be correlated to the increased exposure of the former cohort to the traffic PM pollution than the latter [66]. A study in the Tartu city, Estonia had revealed a strong positive correlation of traffic related PM_2.5_ with the incidence, severity of respiratory and cardiovascular diseases while no significant relation had been observed with pollution related to residential heating [67]. All the observed results from the cytotoxicity, genotoxicity and ROS production assays in this study also substantiated the previous reports and showed the traffic air PM_2.5_ to be more toxic than the PM_2.5_ from other regions (rural, urban, and industrial) which could be related to the presence of high content of metals and PAHs than other samples (Table 4). Hence the further studies on analysis of miRNA and gene expression were constricted to the traffic PM_2.5_ alone. The A549 cells were treated with 50 µg/mL of traffic PM_2.5_ with and without morin pre-treatment and analyzed for the expression of 11 miRNAs and 8 genes related to oxidative stress, inflammation and DNA damage response.

### 3.7. Morin Reverted the Traffic PM_2.5_ Induced Altered miRNA Expression in A549 cells

Though several studies have revealed the protective effect of phytochemicals such as morin against PM_2.5_ related adverse health effects, the exact mechanism behind the action has not yet been clearly elucidated. Recent studies have shown the influence of miRNAs over many cellular mechanisms and certain miRNAs are reported to show altered expression during pathophysiological conditions including inflammation and environmental stress [68] Morin has been shown to reduce the oxidative stress induced DNA damage (genotoxicity) in pancreatic β cell line INS-1E through activation of Nrf2-ARE pathway [37]. MiR155 showed 2-fold over expression on PM_2.5_ associated PAH exposure [69] and miR21 and miR200b were up regulated in lung cancer patients with COPD [70] whereas miR28, miR146a were up regulated in patients with unstable angina and myocardial infarction compared to normal patients [71]. MiR146a and miR21 being negative feedback regulators of secondary inflammatory responses were up regulated in many inflammatory diseases. But miR146a has been suppressed in systemic lupus erythematosus and cigarette smoke exposed cells which show the strong influence of cell type and disease etiology over the miRNA expression in cellular pathology [72]. It has been understood that the adverse health effects of ambient PM_2.5_ might be caused due to the induction of inflammation and oxidative stress that lead to DNA damage [73,74,75]. Hence in this study, 11 miRNAs involved in inflammation and DNA damage response had been tested to ascertain the interaction between miRNA and morin in the cells exposed to traffic PM_2.5_.

**Figure 7:**
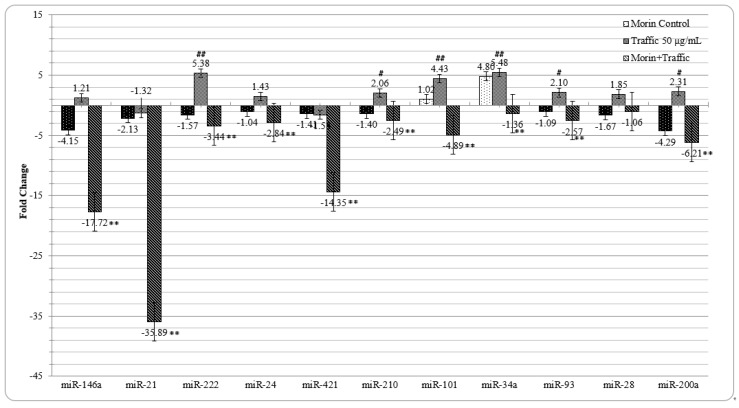
Morin and traffic PM_2.5_ on miRNA expression in A549 cells, N = 3 ± SD. # *p* < 0.05, ## *p* < 0.01 compared to the untreated control cells, ** *p* < 0.01, compared to PM_2.5_-treated cells.

In A549 cells, upon traffic PM_2.5_ exposure, 6 miRNAs (miR222; miR210; miR101; miR34a; miR93; and miR200a) that were reported to be associated to oxidative stress and DNA damage showed more than 2-fold increased expression compared to untreated control cells (Figure 7). In morin pre-treated cells, however, further to these up regulated miRNAs, 4 other miRNAs were also significantly down regulated by more than 2-fold (miR146a; miR21; miR222; miR24, miR421; miR210; miR101; miR93 and miR200a). MiR34a which had 5.48-fold increased expression in traffic PM_2.5_-treated cells was also decreased by −1.36-fold. MiR28 did not have any significant alteration in all three treatment conditions (morin control, traffic PM_2.5_ exposed cells, and morin pre-treated PM exposed cells).

Amid the miRNAs related to the response to DNA damage, miR222 reported to promote cell migration and inhibit apoptosis [76]; miR24 is known to be related to cancer progression; miR210 had been an indicator of hypoxic stress response [77] and known to be involved in DNA damage response [78]; miR93 involved in tumor progression [79] and miR101 had been tumor related miRNAs [80]; miR34a a known oxidative stress responsive tumor suppressor miRNA [26] and miR200a known to induce Nrf2 by inhibiting its suppressor, Keap1. All these 7 miRNAs were found to be up-regulated on traffic PM_2.5_ exposure (miR222: 5.38, miR24: 1.43, miR210: 2.06, miR93: 2.1, miR101: 4.43, miR34a: 5.48, and miR200a: 2.31-fold). From the above miRNA expression pattern, the ability of the traffic PM_2.5_ to elicit DNA damage could be evidently seen. Morin pre-treatment had significantly reduced the expression of all the foresaid miRNAs (miR222: −3.44, miR24: −2.84, miR210: −2.49, miR93: −2.57, miR101: −4.89, miR34a: −1.36, and miR200a: −6.21-fold). MiR-34a as a recognized master regulator of tumor suppression is known to be over expressed in genotoxic stress leading to acetylation of p53 and apoptosis. Its expression is also regulated in a p53-independent manner. For instance expression of miR-34a itself can be affected by inflammatory stimuli [81]. Decreased expression of miR34a observed in morin pre-treated cell may be due to the genoprotective effect of morin or associated with increased expression of NFκB.

**Figure 8:**
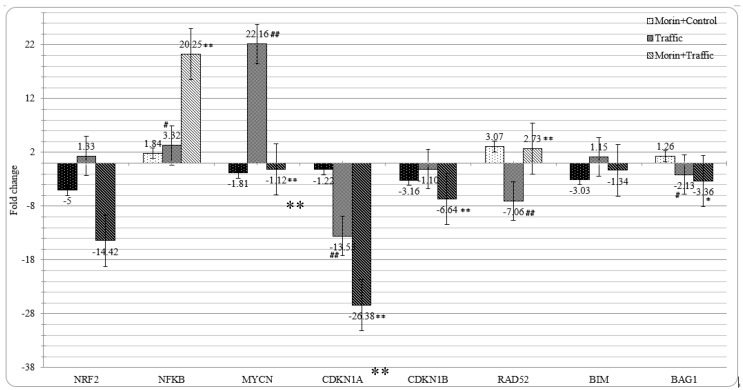
Morin and traffic PM_2.5_ on target gene expression in A549 cells, N = 3 ± SD. # *p* < 0.05, ## *p* < 0.01 compared to the untreated control cells, ** *p* < 0.01, compared to PM_2.5_-treated cells.

Two inflammation related miRNAs, miR146a and miR21 were effectively down regulated by morin pre-treatment (−17.72 and −35.89-fold, respectively). Although these two miRNAs were reported to be up regulated by exposure to metal rich PM_2.5_ [21], here, they were not altered significantly by traffic PM_2.5_ treatment. MiR146a is a dominant negative regulator of NFκB by suppressing TRAF6 that activates IkB kinase (IKK), resulting in degradation of IkB, and nuclear translocation and activation of NFkB [82]. Further, miR146a and miR21 cooperatively suppress secondary inflammation response by decreasing the expression of IL-1β, IL-6, IL-8, IRAK1, MMP-9, and TNF-α [83]. Therefore, enhanced suppression of these miRNAs by morin pre-treatment could be associated to 20.25-fold increase in NFκB expression (Figure 7 and Figure 8).

### 3.8. Morin Revised the Traffic PM_2.5_ Altered Gene Expression in A549 cells

The cellular responses to any external signal would be made in the cells by altered gene expression that might encode for proteins and/or short regulatory RNAs through complex cross-linking signaling pathways mediated by signal mediators and regulatory transcription factors. Oxidative stress mediated Nrf2 pathway and inflammation related NFκB pathway would be the primary cellular response to any stress. Nrf2 had been the key transcription factor for the activation of antioxidant response elements and the production of antioxidant enzymes and cytoprotective enzymes [84]. While exposure to traffic PM_2.5_ did not affect its expression significantly, morin pre-treatment reduced its expression (−14.42-fold). On the other hand, PM_2.5_ exposure increased NFκB expression by 3.32-fold, and morin pre-treatment further increased its expression by 20.25-fold compared to the untreated control cells (Figure 8). NFκB had been known to be up regulated in any cases of stress and immune response [85] and hence it is speculated to be over expressed in any external exposures. Insignificant (*p* < 0.05) reduction was observed in the expression of SOD in A549 cells on exposure to traffic PM_2.5_ (−1.12) and also in cells pre-treated with morin (−1.27) compared to the control untreated cells. These results can be compared with our previous toxicoproteomics study [86] in which PM from steel industry also slightly reduced the SOD levels in A549 cells. MycN had been a known proto-oncogene and elevated levels of MycN had been associated with malignancies. MiR34a and miR9 were reported to be associated with increased MycN levels in non-small lung cancer cells [87]. Traffic PM_2.5_ exposure increased its level in A549 cells by 22.16-fold which could be related to the adverse effect of PM_2.5_ in promoting tumorigenesis. The increased expression of MycN had been significantly reduced in the PM_2.5_ exposed cells pre-treated with morin by −1.12-fold which substantiate the protective effect of morin over the PM induced adverse DNA damage. Further, RAD52, an important protein in RAD51 mediated DNA recombination and repair mechanisms was found to be suppressed (−7.06-fold) in traffic PM_2.5_ exposed cells. However, morin pre-treatment could positively alter the RAD52 expression by 2.73-fold. RAD52 was reported as a target for miR210, as antisense inhibition of miR210 was found to reverse the suppression of RAD52 occurred in hypoxic conditions and restored activation of DNA repair mechanisms [88]. Traffic PM_2.5_ induced up-regulation of miR210 could also be a reason for the traffic PM_2.5_ induced RAD52 down-regulation and as morin pre-treatment suppressed miR210 that could lead into induction of RAD52. Further, over expressing miR222 that could decrease RAD51 expression and efficiency of HR repair [89] and nonhomologous end joining DNA repair inhibiting miR101 [90] in PM_2.5_-treated cells were also inhibited in morin pre-treated cells. These results indicate that traffic PM_2.5_ treatment caused DNA damage and also suppressed DNA repair proteins which were restored by morin pre-treatment.

CDKN1A gene which encodes for cell cycle protein p21 and CDKN1B encoding another cell cycle protein, p27, prevent the cells from entering cell cycle and their down regulation had been associated with the onset of cancers [91]. In this study, traffic PM_2.5_ exposure suppressed CDKN1A expression by −13.55-fold but not CDKN1B (−1.1-fold) expression. Morin pre-treatment failed to revise this change but lead into further suppression (CDKN1A: −26.38 and CDKN1B: −6.64). Since miR222 and miR221 had been observed to regulate CDKN1B gene expression [92], altered expression of miRNAs could be related to the down regulation of CDKN1A and CDKN1B genes. Insignificant over expression of pro-apoptotic protein BIM occurred in traffic PM_2.5_-treated cells was found to be reduced in morin pretreatment. The anti-apoptotic gene BAG1 was down regulated in traffic PM_2.5_ exposure (−2.13-fold) and morin pre-treatment further down regulated its expression by −3.36-fold.

Among four types of PM_2.5_ samples used in this study, traffic PM_2.5_ contained highest levels of metals, PAHs and oxidative potential that could be implicated with higher levels of cytotoxicity and genotoxicity associated with this PM_2.5_ sample. Subsequently, exposure to traffic PM_2.5_ resulted in over expression of DNA damage associated microRNAs. Further traffic PM_2.5_ treatment caused over expression of proto-oncogene MycN and suppression of DNA damage repair gene RAD52. These altered miRNAs and gene expression could occur as a result of traffic PM_2.5_ associated DNA damage and genotoxic stress. Morin pre-treatment revised all these changes in miRNA and gene expression indicating cytoprotective effect of morin. While wide variety of cellular stresses are known to activate Nrf2 and NFκB pathways, these two pathways are proposed to inhibit each other at their transcription level via protein–protein interactions or through secondary messenger effects. Nrf2 pathway inhibits the activation of NFκB pathway by increasing antioxidant defenses and HO-1 expression, which efficiently neutralizes ROS and detoxify toxic chemicals and hence, reduces ROS mediated NFκB activation. Similarly, NFκB mediated transcription reduces the Nrf2 activation by reducing the ARE gene transcription, decreases free CREB binding protein (CBP) by competing with Nrf2 for CH1-KIX domain of CBP. Hence, reduced expression of Nrf2 observed in morin pre-treated cell could be associated to increased levels of NFκB activation.

Based on these observations, traffic PM_2.5_ exposure resulted in oxidative stress and inflammation that leads into increased production of ROS. It also caused DNA damage directly or through ROS. Morin pre-treatment decreased the oxidative stress through its free radical scavenging activity as evidenced by reduced intracellular ROS. However, morin pre-treatment failed to activate Nrf2 which is further reduced by inhibition of miR200a that suppresses Nrf2-suppressor, Keap1. It also appears that morin pre-treatment suppressed miR146a that increased NFκB levels which in turn suppressed already suppressed Nrf2.

## 4. Conclusions

The PM_2.5_ collected from four different regions of Coimbatore city showed different degree of toxicity and traffic PM_2.5_ was found to be more toxic comparatively. Altered miRNA expression profile had always been observed in pathological conditions and PM_2.5_ exposure had significantly altered the expression profile of 11 miRNAs associated with the cellular mechanisms governing PM induced toxicity. Pre-treatment of the A549 cells with the antioxidant morin had effectively provided protection against PM_2.5_ induced toxicity and it also revised the PM_2.5_-induced altered expression of 9 out of 11 miRNAs taken under study. Morin pre-treatment had restored the expression of four genes (Nrf2, NFκB MycN, and Rad52). Hence it can be speculated that the protective effect of morin is mediated through altered oxidative and inflammation associated miRNA expression profile and alteration in expression of Nrf2 and NFκB. Therefore, the protective effect of morin over PM_2.5_ induced genotoxicity can be clearly understood from this study.

## Figures and Tables

**Table 1:** qRT-PCR primers for miRNAs.

miRNA	Forward Primer (5′-3′)
miR 146a (MI0000477)	TGAGAACTGAATTCCATGGGT
miR 21 (MI0000077)	TAGCTTATCAGACTGATGTTGA
miR 222 (MI0000299)	CTCAGTAGCCAGTGTAGATCCT
miR 24 (MI0000080)	TGCCTACTGAGCTGATATC
miR 421 (MI0003685)	ATCAACAGACATTAATTGGGCGC
miR 210 (MI0000286)	AGCCCCTGCCCACCGCACACTG
miR 101 (MI0000103)	CAGTTATCACAGTGCTGATGCT
miR 34a (MI0000268)	TGGCAGTGTCTTAGCTGGTTGT
miR 93 (MI0000095)	CAAAGTGCTGTTCGTGCAGGTAG
miR 28 (MI0000086)	AAGGAGCTCACAGTCTATTGAG
miR 200a (MI0000737)	CATCTTACCGGACAGTGCTG
RNU48	AGTGATGATGACCCCAGGTAA
Universal Reverse Primer for 11 miRNAs (5′-3′)	ATTCTAGAGGCCGAGGCGGCCGACATGT

**Table 2:** qRT-PCR primers for the target genes.

Target Gene	Forward Primers (5′-3′)	Reverse Primers (5′-3′)
NrF2	ATGCCCTCACCTGCTACTTT	GCCAAGTAGTGTGTCTCC
NFκB	ATCCTTCTTTGACTCATACA	CCTTTGCTGGTCCCACATAG
MycN	AAGCCCTGGACGGGATTGCG	CGCAGAAGCCATTACTCCC
CDKN1A	TGGGGATGTCCGTCAGAACC	CCTCCTCCCAACTCATCCCG
CDKN1B	CCGCCCTCCCGCTCGCCAG	GTCCATCCGCTCCAGGCTA
RAD52	CTCCCACCTCTGCCTTACAA	CCCATCCCCAAGGTCTCATT
BIM	TGACTCTGACTCTCGGACTG	TCCAATACGCCGCAACTCT
BAG1	TGGGAAGTAGTCGGGCGGGG	CGAGAGGGAGGCGGACCACG
β-actin	GGCGGACTATGACTTAGTTG	AAACAACAATGTGCAATCAA

**Table 3:** Characteristics of fine particulate matter (PM_2.5_) samples.

PM_2.5_ Sampling Area	Concentration of PM_2.5_ (µg/m^3^ of air)	PM_2.5_ Size Range (nm)	Peak Intensity Size (nm)	Polydispersity Index (PDI)	Zeta Potential (mV)	Conductivity (mS/cm)
Rural	30.34	750–2585	759.4	1.000	−34.7	0.021
Urban	36.53	550–2330	552.9	1.000	−36.6	0.043
Industrial	41.93	440–1060	444.0	0.962	−29.5	0.113
Traffic	51.47	605–1980	605.1	1.000	−31.0	0.023

**Table 4:** Concentration of metals (ng/m^3^ of air) in PM_2.5_ samples.

	Rural	Urban	Industrial	Traffic
As	0.003	0.006	0.009	0.002
Cd	-	0.001	-	-
Cr	0.057	0.065	0.628	0.056
Cu	0.001	0.001	0.001	0.001
Fe	0.985	1.116	1.699	1.357
Mn	0.039	0.048	0.006	0.029
Ni	0.001	0.002	0.001	0.002
Se	0.001	0.001	0.002	0.001
Zn	0.001	0.002	0.004	0.003
Al	0.004	0.002	0.019	0.002
V	0.003	0.008	0.012	0.005
Pb	0.228	0.322	0.318	0.710
Total metal	1.323	1.574	2.696	2.167

**Table 5:** Concentration of polycyclic aromatic hydrocarbons (PAHs) (ng/m^3^ of air) in PM_2.5_ samples.

Name of PAH	Rural	Urban	Industrial	Traffic
Total PAH	5.6	20.1	25.0	24.8
*NaP*	-	-	-	0.2
*Acy*	-	-	-	0.7
*Ace*	-	-	0.2	1
*Fl*	-	-	0.1	-
*Phe*	1.6	-	3.6	-
*Ant*	0.2	-	3	1.4
*Flu*	0.8	0.4	11.5	0.5
*Pyr*	-	1.4	4.1	1.8
*BaA*	-	3.6	-	2.8
*Chr*	-	7.6	-	8.2
*BbF*	-	3.2	-	3.9
*BkF*	-	-	-	0.1
*BaP*	1.5	2.6	1.8	2.5
*DBA*	1.5	1.3	0.7	1.7
*BghiP*	-	-	-	-
*IND*	-	-	-	-

Detection Limit is 0.5 ng/m^3^ of air. Abbreviations: Napthalene (NaP), Acenapthylene (Acy), Acenapthene (Ace), Fluorene (Fl), Phenanthrene (Phe), Anthracene (Ant), Fluoranthene (Flu), Pyrene (Pyr), Benzo(a)anthracene (BaA), Chrysene (Chr), Benzo(b)fluoranthene (BbF), Benzo(k)fluoranthene (BkF), Benzo(a)pyrene (BaP), Dibenzo(a,h)anthracene (DBA), Benzo(ghi)pyrene (BghiP), and Indeno(123-cd)pyrene (IND).
